# A novel *TFF2* splice variant *(ΔEX2TFF2)* correlates with longer overall survival time in cholangiocarcinoma

**DOI:** 10.3892/or.2011.1583

**Published:** 2011-12-07

**Authors:** SURASEE KAMLUA, SIRIPORN PATRAKITKOMJORN, PATCHAREE JEARANAIKOON, TREVELYAN R. MENHENIOTT, ANDREW S. GIRAUD, TEMDUANG LIMPAIBOON

**Affiliations:** 1 Graduate School, Khon Kaen University, Khon Kaen 40002, Thailand; 2 Centre for Research and Development of Medical Diagnostic Laboratories, Faculty of Associated Medical Sciences, Khon Kaen University, Khon Kaen 40002, Thailand; 3 Liver Fluke and Cholangiocarcinoma Research Center, Faculty of Medicine, Khon Kaen University, Khon Kaen 40002, Thailand; 4 Murdoch Children’s Research Institute, Royal Children’s Hospital, Parkville, VIC 3052, Australia

**Keywords:** cholangiocarcinoma, trefoil factor 2, alternative splicing mRNA, biomarker

## Abstract

Trefoil factor 2 (TFF2) is a member of trefoil factor family found to be overexpressed in many cancers including cholangiocarcinoma (CCA). The majority of studies have focused on wild-type TFF2 (wtTFF2) expression, but information regarding alternative splicing variants of *TFF2* mRNA has not been reported. In this study, we aimed to identify and quantify a novel *TFF2* splice variant in cholangiocarcinoma (CCA). Seventy-eight tumors and 15 normal adjacent tissues were quantified for the expression of the *TFF2* splice variant relative to wild-type (wt) *TFF2* mRNA using quantitative reverse transcriptase polymerase chain reaction (QRT-PCR). The ratio of *TFF2* splice variant against *wtTFF2* was analyzed for associations with clinical parameters. We found a novel *TFF2* splice variant, exon 2 skipping *(ΔEX2TFF2)*, resulting in a stop codon (TAG) at exon 1. The *ΔEX2TFF2/wtTFF2* ratio in tumors was significantly higher than in normal tissue (P<0.01). Interestingly, high *ΔEX2TFF2/wtTFF2* ratio was significantly associated with good prognosis compared with low ratio (P=0.017). In contrast, the presence of wtTFF2 protein was associated with poor survival of CCA patients (P=0.034). This is the first report of a trefoil factor splice variant and its potential application as a prognostic biomarker in CCA.

## Introduction

Cholangiocarcinoma (CCA) is a malignancy of bile duct epithelia and the most common liver cancer in Northeast Thailand. CCA is responsible for 71% of liver cancer in Khon Kaen, representing the highest incidence of CCA in the world (1). Epidemiological and animal studies showed the association of CCA development with liver fluke *(Opisthorchis viverrini)* infection and carcinogenic nitrosamine (1–4). The survival rate of CCA patients is low because most patients are diagnosed at late stage of the disease. Prognosis of CCA is poor and the majority of patients die within 6–12 months after diagnosis. The relative survival of liver cancer in Khon Kaen is 23.2% for one year, 11.1% for three years, and 8.4% for five years (5). Although the molecular mechanisms for the development of liver fluke-associated CCA have been widely studied in the past decade, the clearer image of how the studied genes mediate carcinogenesis and pathogenesis of this disease remains obscure (6,7).

The trefoil factor *(TFF)* is a cluster of gene located on chromosome 21q22.3 encoding polypeptides TFF1, TFF2, and TFF3. TFF proteins are well known for their potent protective and healing effects termed ‘restitution’ after mucosal damage in the gastrointestinal tract. To achieve their role in epithelial restitution, TFFs protect cells from apoptotic death and stimulating cell migration (8,9). The deregulation of these functions contributes to TFF-mediated cancer development and progression (10–12). Our previous study showed that the expression of *TFF2* mRNA in CCA was significantly higher than in normal tissues (13). Moreover, TFF2 positive immunostaining in CCA was markedly increased compared with normal and precancerous tissues suggesting its important role in tumor progression (13). In addition, we also demonstrated that TFF2 activates proliferation of CCA cells via epidermal growth factor receptor (EGFR) and mitogen-activated protein kinase (MAPK) signaling (13). While TFF2 and its role in CCA have been defined, we raised the question whether there are splice variants of *TFF2* existed and whether these isoforms have adversely affected on patient prognosis and tumor progression since some tumors sustain their malignant behavior by aberrant expression of splice variants (14). Likewise, the presence of splice variants of many genes i.e. *p53* gene family has been shown to be associated with poor patient outcomes (15,16).

In this study, we investigated the occurrence of a novel alternatively spliced variant of the *TFF2* gene in CCA and quantified the splicing isoform *TFF2* in CCA and normal adjacent tissues. The ratio of splicing isoform and wild-type *TFF2* was analyzed for associations with clinical parameters. In addition, correlation between expression of alternatively spliced variant of *TFF2* mRNA and wild-type TFF2 protein expression was also tested.

## Materials and methods

### Cholangiocarcinoma (CCA) samples

Six CAA cell lines including KKU-OCA17, KKU-100, KKU-M055, KKU-M139, KKU-M156 and KKU-M213 kindly provided by the Liver Fluke and Cholangiocarcinoma Research Center (LFCRC), Faculty of Medicine, Khon Kaen University were cultured in HAM-F12 supplemented with 10% fetal bovine serum (FBS). The cells were maintained at 37°C in a humidified atmosphere with 5% CO_2_ and 80–100% confluence of cell lines were subpassaged or harvested by trypsinization.

Seventy-eight samples were obtained from resected tissues of CCA patients undergoing surgery at Srinagarind Hospital, Faculty of Medicine, Khon Kaen University. Signed informed consents were received from all patients participating in this research project. This study was approved by the Khon Kaen University Ethics Committee for Human Research (HE522133). All resected tissues were stored at −70°C until used. Histology was routinely examined and the clinical data of the patients were collected.

### RNA isolation and complementary DNA (cDNA) preparation

Total RNA was extracted from fresh frozen CCA tissues and CCA cell lines with RNeasy kit (Qiagen, Valencia, CA, USA) according to the manufacturer’s instructions. RNA was quantified using GE NanoVue spectrophotometer (GE Healthcare, Buckinghamshire, UK). For reverse transcription, oligo-dT primed first strand cDNA was synthesized from 1 μg template RNA using Improm-II™ reverse transcription system kit (Promega, Madison, WI, USA) according to the manufacturer’s instructions.

### Reverse transcription-polymerase chain reaction (RT-PCR)

To screen the expression pattern of *TFF2* isoform mRNA, RT-PCR was performed with cDNA derived from CCA cell lines and tumor tissues. The sequences of full length primers are shown in Table I. The expected amplified product size is 434 base pairs for *wtTFF2* and 284 base pairs for splicing isoform of *TFF2*. PCR products were selected for nucleotide sequencing to verify type of splicing isoform of *TFF2* and to construct the plasmid clones used as a standard in quantitative reverse transcription-PCR (QRT-PCR) method.

### Quantitative reverse transcription-PCR (QRT-PCR)

QRT-PCR was performed to quantify the level of gene expression of *wtTFF2*, splicing isoform *TFF2 (ΔEX2TFF2)* and *GAPDH* (reference gene), using a SYBR Green I assay (Amresco, Solon, OH, USA). The sequences of these primers are summarized in Table I. The *GAPDH* primer sequences are given elsewhere (17). PCR was conducted on a Rotor-Gene 6 (Corbett Research, Australia) and a melting curve profile was analyzed for specific product. Copy number of gene expression was generated from the standard curve, using plasmid clones as DNA standards; copy number ranging 30-3×10^6^ for *wtTFF2* and *ΔEX2TFF2*, and 3×10^2^-3×10^7^ for *GAPDH*. Each sample was run in triplicate and the coefficient of variation (CV) <15% was acceptable.

The relative expression of mRNA was normalized with *GAPDH* before the ratio of *ΔEX2TFF2/wtTFF2* was obtained. The *ΔEX2TFF2/wtTFF2* ratio was categorized into low and high expression based on the cut-off value derived from corresponding normal controls (cut-off value = upper quartile value [75th percentile] + 1.5 [interquartile range]). Association of *ΔEX2TFF2/wtTFF2* ratio was analyzed with clinicopathological data.

### Western blotting

Total protein was extracted from CCA samples using TRIzol^®^ reagent (Invitrogen, Carlsbad, CA) according to the manufacturer’s protocol. Protein was fractionated on 15% SDS-PAGE and transferred to a parablot Polyvinylidine Fluoride (PVDF) membrane (MN, Germany). The membrane was non-specifically blocked with 5% skim milk in Tris Buffered Saline (TBS) (150 mM NaCl, 50 mM Tris, pH 7.5) for 1 h, then incubated with primary antibody at dilution 1:3000, 4°C overnight. Sources of primary antibodies: rabbit polyclonal anti-human TFF2 (A gift from Professor Andrew Giraud, Murdoch Children’s Research Institute, Royal Children’s Hospital, Parkville, Australia); rabbit polyclonal to β-actin (Abcam, Cambridge, UK). After washing, a dilution 1:3000 of secondary antibody, goat polyclonal to rabbit IgG conjugated with horseradish peroxidase (Abcam, Cambridge, UK), was applied and the immune complexes were detected by an enhanced chemiluminescence (ECL) system (GE Healthcare). The band intensity was analyzed using Image J software distributed by the National Institutes of Health (http://rsb.info.nih.gov/ij/index.html). The expression of wtTFF2 was quantitated by comparison with standard 100 nanogram of glycosylated-recombinant human TFF2 (rhTFF2) (A gift from Professor Andrew Giraud).

The wtTFF2 protein expression was categorized into low and high expression based on the cut-off value derived from corresponding normal controls [cut-off value = upper quartile value (75th percentile) + 1.5 (interquartile range)]. The expression of wtTFF2 was analyzed for the association with clinicopathological data.

### Statistical analysis

The difference of splice variant of *TFF2* mRNA expression in normal and tumor tissues was analyzed using Mann-Whitney test. Correlations between splice variant of *TFF2* mRNA and *wtTFF2* protein expression were analyzed using Spearman’s rho test. Associations of splice variant mRNA and wtTFF2 protein expression with clinicopathological data of CCA patients were evaluated using χ^2^ test. Survival analysis was analyzed using Kaplan Meier and log rank test. P<0.05 was considered as statistical significance. All statistical analyses were performed using SPSS.

## Results

### Expression pattern of TFF2 mRNA isoforms in CCA

**Figure 1:**
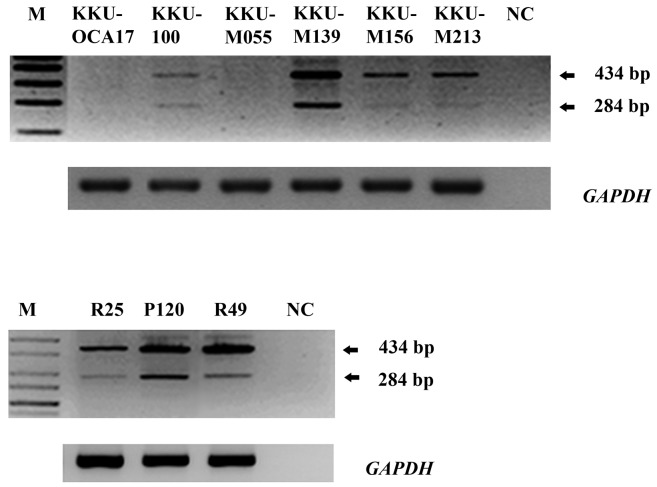
Expression pattern of *TFF2* mRNA in CCA detected by RT-PCR. The full-length of 434 bp and a smaller fragment of 284 bp were detected in both (A) CCA cell lines (4/6) and (B) CCA samples (3/3). NC, negative control; bp, base pair; M, Size marker.

**Figure 2:**
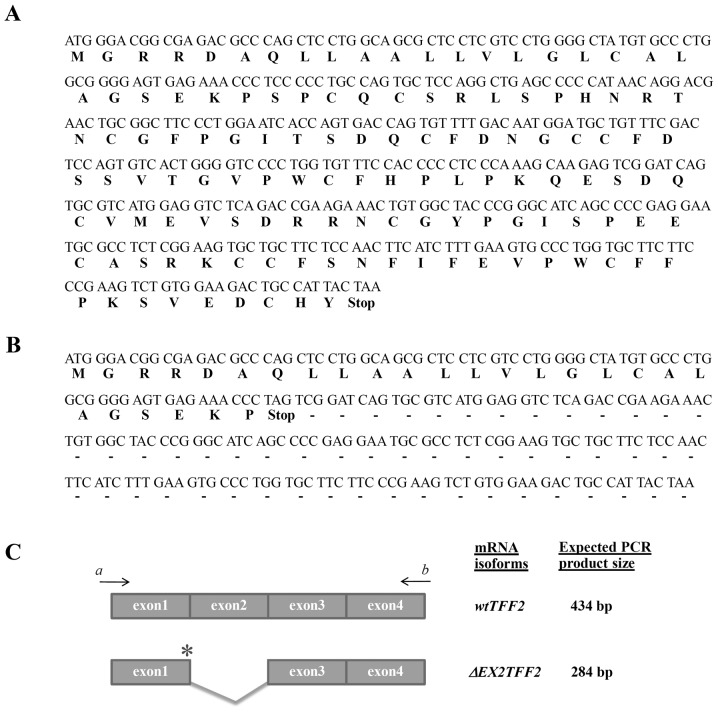
Nucleotide sequence of full length *TFF2* and splicing isoform with exon 2 skipping *(ΔEX2TFF2)*. (A) Nucleotide sequence of full length *TFF2* mRNA with 129 amino acid sequence. (B) Nucleotide sequence of *ΔEX2TFF2* with predicted 26 amino acid sequence. (C) Schematic diagram of alternative splicing isoform of *TFF2* revealed an out-of-frame skipping of exon 2. Arrow lines, locations of sense *(a)* and antisense *(b)* primers. *, premature termination codon found in exon 1; bp, base pair.

Six CCA cell lines including KKU-OCA17, KKU-M055, KKU-100, KKU-M139, KKU-M156 and KKU-M213 were determined for *TFF2* mRNA expression by RT-PCR which could detect both full length and splicing isoform. The PCR products revealed the *TFF2* full length (434 bp) and a smaller (284 bp) fragment. Four out of six CCA cell lines, KKU-100, KKU-M139, KKU-M156 and KKU-M213, exhibited both sizes, which were also detected in CCA samples (Fig. 1). Nucleotide sequencing was performed and showed exon 2 skipping *(ΔEX2TFF2)* resulting in a premature termination codon (PTC) within exon 1 in which only signal sequence was obtained (Fig. 2).

### Expression of ΔEX2TFF2 in CCA samples. ΔEX2TFF2

mRNA was quantified in 78 CCA and 15 normal adjacent tissues using QRT-PCR. The median relative expression of *ΔEX2TFF2* mRNA in normal adjacent and CCA tissues were 0.00 (min: 0.00, max: 4.29×10^−5^) and 8.98×10^−5^ (min: 0.00, max: 1.80×10^−2^), respectively. The median of *ΔEX2TFF2/wtTFF2* ratio in normal adjacent and CCA tissues were 0.00 (min: 0.00, max: 2.35×10^−2^) and 0.11 (min: 0.00, max: 17.47), respectively.

**Figure 3:**
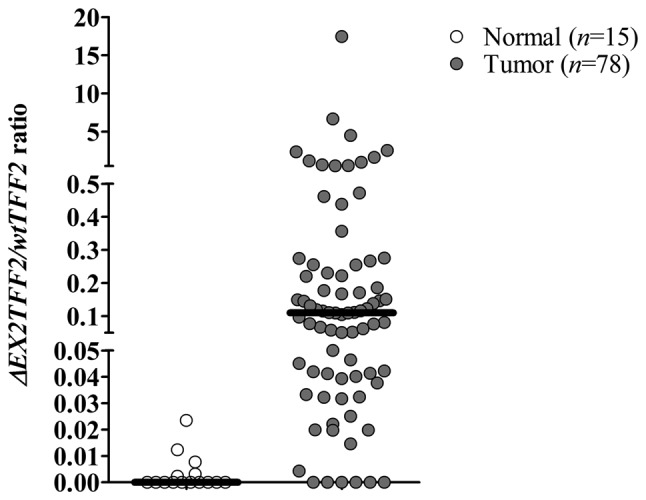
Ratio of *ΔEX2TFF2/wtTFF2* in tumor tissues compared with normal adjacent tissues. There was a significant difference between *ΔEX2TFF2/wtTFF2* ratio of normal adjacent tissues and tumor tissues (P<0.01). Horizontal black bars show median ratio.

**Figure 4:**
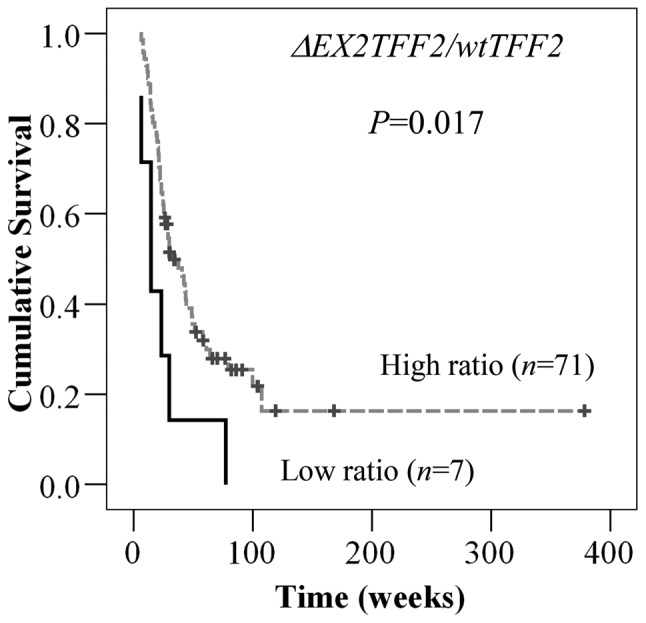
Survival curves of CCA patients with *TFF2* isoform expression. Patients with high ratio of *ΔEX2TFF2/wtTFF2* displayed longer overall survival than patients with low ratio (P=0.017).

It has been shown that *TFF2* is involved in tumor progression in CCA (13). Accordingly, alternative splicing may be one mechanism by which cells employ to regulate the expression of *TFF2* in term of transcript and protein level. We then postulated that alternatively spliced variant of *TFF2* may regulate the expression of the wild-type (wt) *TFF2* in a negative feedback fashion and the expression of *ΔEX2TFF2* may be a good prognostic indicator in CCA patients. We analyzed the median of *ΔEX2TFF2/wtTFF2* ratio between CCA and normal adjacent tissues and found the significant difference between these two groups (P<0.01, Fig. 3). To analyze associations between *ΔEX2TFF2/wtTFF2* ratio and clinicopathological data of CCA patients, we categorized *ΔEX2TFF2/wtTFF2* ratio into low and high expression based on the cut-off value, as described above (cut-off = 6.93×10^−3^). As expected, patients with high ratio of *ΔEX2TFF2/wtTFF2* had longer overall survival than patients with low ratio (P=0.017, Fig. 4). However, there were no associations of *ΔEX2TFF2/wtTFF2* ratio with age, gender and histological grading.

### Wild-type TFF2 protein expression in CCA

**Figure 5:**
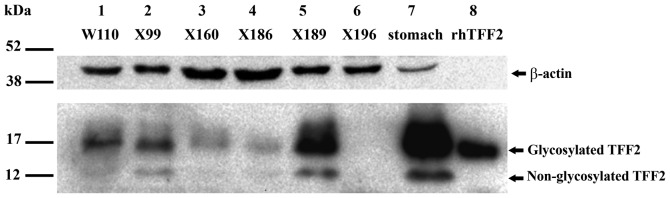
Representative expression of wtTFF2 protein in CCA tissues using western blotting. Total protein ~20 μg was separated in a 15% SDS-PAGE. Upper panel showed β-actin expression in CCA samples (Lanes 1–6) and stomach (Lane 7). Lower panel showed wtTFF2 expression, the glycosylated wtTFF2 was 17 kDa while non-glycosylated form was 14 kDa. Wild-type TFF2 protein was expressed in CCA samples (Lanes 1–5) except sample no. X196 (Lane 6). Both glycosylated and non-glycosylated wtTFF2 were found in normal stomach tissue (Lane 7). Lane 8, recombinant human TFF2 (100 ng).

**Figure 6:**
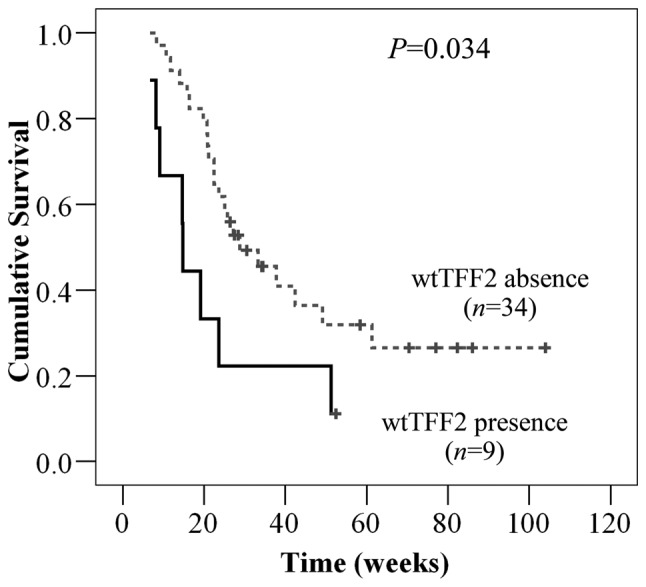
Survival curves of CCA patients with presence and absence of wtTFF2. Presence of wtTFF2 was associated with the decreased overall survival time of CCA patients (P=0.034).

**Figure 7:**
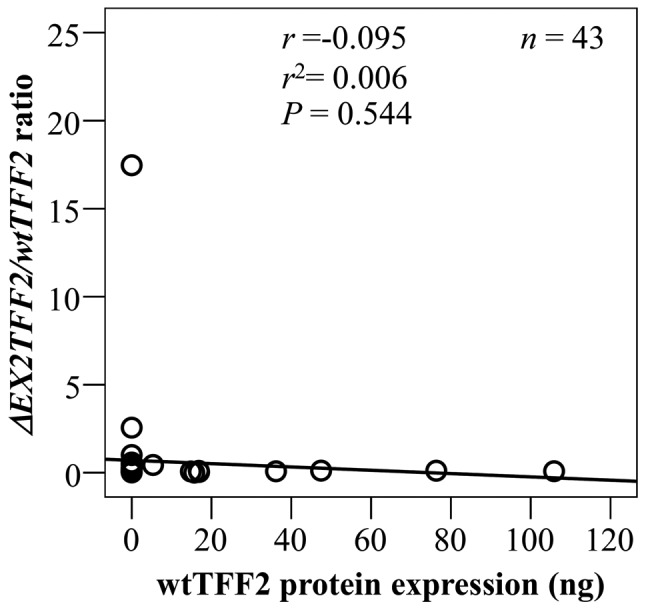
Scatter plot of correlation between *ΔEX2TFF2/wtTFF2* ratio and wtTFF2 protein expression in CCA. No significant correlation was found.

Expression of wtTFF2 protein was quantitated in 43 CCA tissues using western blotting. Recombinant human (rh) TFF2 and protein derived from gastric tissue were used as a positive control for TFF2 protein expression. The result showed that wtTFF2 protein, glycosylated and non-glycosylated TFF2 (Fig. 5), was found in 9/43 CCA and 1/15 normal adjacent tissues. To analyze the association between wtTFF2 protein expression and clinocopathological data of CCA patients, the expression of wtTFF2 protein was categorized into low and high expression based on a cut-off value, as described above. The cut-off value was 0.00. Thus, we categorized it into two groups, the presence and absence of wtTFF2. The presence of wtTFF2 was significantly associated with the decreased overall survival time in CCA patients (P=0.034, Fig. 6) suggesting its important role in tumor progression. However, the presence of wtTFF2 was not associated with age, gender and histological grading. As postulated previously regarding the effect of splice variant on TFF2 protein expression, we tested the correlation between *ΔEX2TFF2/wtTFF2* ratio and wtTFF2 protein expression. The results showed that *ΔEX2TFF2/wtTFF2* ratio was not significantly correlated with wtTFF2 protein level but it had a trend negative correlation (r= −0.095, P=0.544, Fig. 7).

## Discussion

We are the first to show the presence of an alternatively spliced variant, exon 2 skipping, in CCA. This type of splice variant, *ΔEX2TFF2*, resulted in a premature termination codon (PTC) in the exon 1 in which only signal sequence was encoded. The non-functional TFF2 cannot mediate cell proliferation like its canonical TFF2 preventing tumor cell progression. Our findings supported this postulation in which good prognosis was found in patients with high *ΔEX2TFF2/wtTFF2* ratio. Thus, *ΔEX2TFF2/wtTFF2* ratio may serve as a prognostic factor of good outcome in CCA. Moreover, the mRNA with PTC subjected to be degraded by the nonsense mediated decay (NMD) pathway (18,19) has been shown to have a very short half-life. The presence of splice variant of *TFF2* in CCA patients suggested that alternative splicing may be a process which regulates the expression of *TFF2*. Therefore, it is conceivable that the overexpression of splice variant may play a pivotal role as a dominant inhibitor of wild-type function. In this study, we could not find a significant inverse correlation between the *ΔEX2TFF2/wtTFF2* ratio and wtTFF2 protein (r= −0.095) suggesting that there are other mechanisms rather than alternative splicing which control the expression of TFF2. Further study is needed to address whether *ΔEX2TFF2* plays a role in translational regulation of wtTFF2 protein.

Our recent study reported that TFF2 positive immunostaining was markedly increased in CCA compared with those in normal bile ducts and dysplasia suggesting the role of TFF2 in tumor progression (13). In this study, we demonstrated that the expression of wtTFF2 protein was associated with poor prognosis of CCA patients. This is consistent with the previous studies which demonstrated that high expression of TFF2 was an independent indicator of poor prognosis in CCA (20) and gastric cancer patients with TFF2-expressing tumors had a significantly worse disease-free survival (11,21). Thus, wtTFF2 expression may serve as a prognostic marker of poor outcome in CCA.

In conclusion, we identified a novel *TFF2* splice variant, *ΔEX2TFF2*, and its significance in term of *ΔEX2TFF2/wtTFF2* ratio, which was upregulated in CCA. The splice variant *ΔEX2TFF2* might act as a negative regulator of wtTFF2 expression. Clinically, the high ratio of *ΔEX2TFF2/wtTFF2* serves as a potential prolonged survival marker of CCA patients. In contrast, the presence of wtTFF2 protein is strongly associated with shorter overall survival time. This study suggests the use of wtTFF2 inhibitor as a targeted therapy for effective treatment of CCA.

## Figures and Tables

**Table I:** Primer sequences used for detection of TFF2 gene expression.

Primer name	Forward (5′→3′)	Reverse (5′→3′)	Product size (bp)
RT-PCR			
*FL-TFF2*	TGCAGCTGAGCTAGACATGG	CAGATGCATCCTCTGGAACC	434
QRT-PCR			
*wtTFF2*	TTCCCTGGAATCACCAGTGACC	ATGACGCACTGATCCGACTCTTG	119
*ΔEX2TFF2*	CGGGGAGTGAGAAACCCTAGT	CCACAGACTTCGGGAAGAAGC	162
